# Transcriptional control of stem cell fate by E2Fs and pocket proteins

**DOI:** 10.3389/fgene.2015.00161

**Published:** 2015-04-28

**Authors:** Lisa M. Julian, Alexandre Blais

**Affiliations:** ^1^Sprott Centre for Stem Cell Research, Regenerative Medicine Program, Ottawa Hospital Research Institute, Ottawa, ONCanada; ^2^Ottawa Institute of Systems Biology, Ottawa, ONCanada; ^3^Department of Biochemistry, Microbiology, and Immunology, Faculty of Medicine, University of Ottawa, Ottawa, ONCanada

**Keywords:** stem cell fate, neural precursor cell (NPC), pocket proteins, transcription, epigenetics, stem cells, cell cycle, E2F transcription factors

## Abstract

E2F transcription factors and their regulatory partners, the pocket proteins (PPs), have emerged as essential regulators of stem cell fate control in a number of lineages. In mammals, this role extends from both pluripotent stem cells to those encompassing all embryonic germ layers, as well as extra-embryonic lineages. E2F/PP-mediated regulation of stem cell decisions is highly evolutionarily conserved, and is likely a pivotal biological mechanism underlying stem cell homeostasis. This has immense implications for organismal development, tissue maintenance, and regeneration. In this article, we discuss the roles of E2F factors and PPs in stem cell populations, focusing on mammalian systems. We discuss emerging findings that position the E2F and PP families as widespread and dynamic epigenetic regulators of cell fate decisions. Additionally, we focus on the ever expanding landscape of E2F/PP target genes, and explore the possibility that E2Fs are not simply regulators of general ‘multi-purpose’ cell fate genes but can execute tissue- and cell type-specific gene regulatory programs.

## Introduction

Since the discovery of the retinoblastoma protein (pRb) as a potent tumor suppressor two and a half decades ago, the pocket protein (PP) family (including pRb, p107 and p130) and their best characterized interacting partners, the E2F transcription factor family, have been under intensive scientific investigation. It is now clear that the PP and E2F proteins are not only important regulators of cellular proliferation but of multiple cellular processes, many of which impact cell fate decisions. While cell cycle-independent roles for E2Fs and PPs have been known for some time (Lee et al., 1994), what remains to be fully clarified, however, are the mechanisms by which the PP and E2F families control such diverse functions.

The advent of genomics and other systems biology approaches to study the role and mode of action of transcription factors has contributed greatly to our mechanistic understanding of E2F/PP function. Additionally, work by many groups, predominantly over the past decade, focused on linking causative target genes to non-canonical E2F/PP biological functions has greatly enriched our view of E2Fs and PPs as regulators of not only cell cycle control, but also key cell fate decisions. Collectively, these studies suggest that E2fs and PPs are dynamic transcriptional regulators that can control diverse cellular functions by regulating genes directly involved in those processes, potentially in a highly tissue-specific manner.

In this review we discuss the current understanding of how the classical cell cycle regulatory pathway impacts cell fate decisions at the level of E2F/PP-dependent transcriptional regulation. Specifically, we highlight findings that position E2F and PP factors as fundamental regulators of cell fate control in stem and progenitor populations. Furthermore, we discuss emerging mechanisms by which E2Fs and PPs may execute cell type-specific gene regulatory programs in order to regulate cell fate control in a specialized manner.

### Cell Cycle Regulation by E2Fs and PPs

The eukaryotic cell cycle is controlled in large part by the cyclical expression of important effector molecules. For example, the expression of enzymes that participate in DNA replication or chromosome segregation typically occurs when these proteins are needed, in S or M phase, respectively. While a great deal of regulation of this process occurs at the level of controlled synthesis-degradation of certain regulatory proteins (most notably the cyclins), transcriptional control by sequence-specific E2F transcription factors and their regulation by PPs is also heavily implicated as a central mechanism driving cell cycle regulation [reviewed in Dick and Rubin (2013)].

To date, eight *E2F* genes, giving rise to 10 distinct E2F proteins, have been identified in mammals [reviewed in Chen et al. (2009b)]. While E2F factors exhibit varying degrees of sequence and structural differences, the DNA binding domain is strikingly well-conserved among family members. This befits findings that E2F family members typically exhibit significant overlap in their target genes in a given tissue (Xu et al., 2007). The classical view of E2F/PP activity in cell cycle control (Cam and Dynlacht, 2003) is that unphosphorylated PPs form transcriptional repressive complexes with repressor E2Fs (E2F3b, E2F4, and E2F5) in quiescent and early G1 phase cells, to silence the expression of cell cycle regulatory and effector genes. In the presence of mitogenic stimuli, cyclin D-CDK4/6 initiates the phosphorylation of PPs, which leads to the disruption of the E2F/PP repressive complexes and nuclear export of the E2F factors. Concomitantly, activator E2F proteins (E2F1, E2F2, and E2F3) become expressed and stimulate the transcription of cell cycle genes that allow cells to pass the G1/S transition.

### An Expanded Role for E2Fs and PPs in Controlling Stem and Progenitor Cell Fate Decisions

As a central regulator of proliferation and cell cycle exit, the E2F/PP pathway is functional in essentially all cell types, and during all stages of development. Investigations into the biological roles of cell cycle regulatory proteins beyond fibroblasts and tumor-derived cell lines, specifically within tissue-specific primary stem and progenitor cell populations, have revealed that this pathway controls a number of cellular processes, many of which impact key stem cell fate decisions. This is exemplified collectively by findings that loss of pRb and/or the other PPs results in stem cell expansion in many tissues, often accompanied by decreased cell survival, inhibition of differentiation, or altered lineage choices upon differentiation [reviewed in Sage (2012), Cai et al. (2013), De Sousa et al. (2014)]. Deregulation of E2F activity is strongly implicated in driving many of these phenotypes, and the existing literature now suggests a fundamental widespread role for these transcriptional regulators in cell fate determination.

Similar to the strong evolutionary conservation of a role in cell cycle regulation (Dimova et al., 2003; Stevaux et al., 2005; Kirienko and Fay, 2007; Hirano et al., 2008; Acharya et al., 2012; Korenjak et al., 2012; Kudron et al., 2013), E2F/PP-mediated control of stem cell fate decisions also appears to be deeply conserved. The PP and repressive E2F orthologs in the highly regenerative freshwater planarian (*Smed-Rb* and *Smed-E2F4-1*, respectively) are required for the self-renewal, maintenance and survival of pluripotent adult stem cells in this system (Zhu and Pearson, 2013). Additionally, a clear role for E2Fs and PPs in regulating stem cell fate decisions was in fact first demonstrated in the plant species *Arabidopsis thaliana*. In this system, functional suppression of the single PP RBR or over-expression of the transcriptional activator E2Fa leads to a specific increase in the number of stem cells in the root meristem; conversely, *RBR* over-expression causes these cells to rapidly differentiate (Wildwater et al., 2005). *RBR* loss also results in an expanded stem cell pool and aberrant fate determination in the male germline (Chen et al., 2009c).

## A Multi-Tissue Cell Fate Regulatory Role for E2F and Pocket Proteins

The earliest indications that the functional importance of the cycle machinery extends beyond the regulation of cell cycle progression in mammalian systems came from analysis of *Rb1* knockout mice. *Rb1*-deficient embryos die between embryonic day 13.5–15.5 and they are marked by ectopic mitoses and extensive apoptosis throughout the developing nervous system (Clarke et al., 1992; Jacks et al., 1992; Lee et al., 1992; Morgenbesser et al., 1994). This demonstrated a potential novel role for pRb in cell survival. These and subsequent studies additionally revealed an essential role for pRb in cell cycle exit and cellular differentiation, predominantly within the myoblast, neural, erythroid, and trophoblast stem cell lineages (Clarke et al., 1992; Jacks et al., 1992; Lee et al., 1992, 1994; Slack et al., 1998; de Bruin et al., 2003; MacPherson et al., 2003; Wu et al., 2003). The aberrant trophoblast stem cell differentiation induced by pRb loss was later attributed to deregulated E2f3 activity (Wenzel et al., 2007) and antagonism between the E2f3 and E2f7&8 factors (Ouseph et al., 2012). Further, conditional loss of pRb in muscle precursors (Rb-flox:Myf5-Cre) led to a reduced differentiation capacity and increased rates of apoptosis (Huh et al., 2004), demonstrating the cell autonomous nature of these effects. Together, these phenotypic studies suggested an essential role for pRb in embryonic development and post-natal survival, characterized by widespread roles in cellular proliferation, survival, and differentiation.

Deficiency in PPs other than pRb revealed additional roles for this family in differentiation and survival. Compound deficiency for both pRb and either p107 or p130 results in phenotypes similar to *Rb1* knockouts, but these mice die earlier and display an exacerbation of proliferative and apoptotic phenotypes in a number of tissues, including the central nervous system (CNS; Lee et al., 1996; Lipinski and Jacks, 1999; Sage et al., 2000; Berman et al., 2009). Mice lacking both p107 and p130 also exhibit perinatal lethality and have defects in chondrocyte and epidermal differentiation (Cobrinik et al., 1996; Ruiz et al., 2004). Finally, loss of all three PPs demonstrated an essential role in early development and pluripotency, as these mice die by E9.5-11.5 with evidence of widespread elevated proliferation and cell death (Wirt et al., 2010). Furthermore, triple PP-deficient human embryonic stem cells (ESCs) exhibit cell cycle arrest and death, by activation of p53 and p21 signaling (Conklin et al., 2012). Thus, loss of PPs leads to marked defects in development and differentiation of many cell and tissue types.

In the tumor prone retina, pRb is required in a cell autonomous manner for progenitor cell exit and differentiation of rod photoreceptor cells (Zhang et al., 2004), while the PP family is together required to maintain horizontal interneurons in a post-mitotic state (Ajioka et al., 2007). In the absence of PPs, horizontal cells maintain their differentiated state but begin to clonally expand, giving rise to metastatic retinoblastomas. pRB loss in human retinal cone cells has also been demonstrated to drive cell cycle exit and to promote retinoblastoma-like tumor development (Xu et al., 2014). E2fs themselves are also heavily involved in the proliferation, survival, and differentiation of distinct neuronal cell types in the retina (Chen et al., 2007, 2013). Additionally, E2F1 and hyper-phosphorylated pRB play important roles in post-mitotic neurons in the adult brain, specifically in effecting the calpain-induced neuronal cell death observed in a number of CNS neurocognitive disorders, including HIV-induced encephalitis, Alzheimer disease, Parkinson’s disease, and amyotrophic lateral sclerosis (Giovanni et al., 2000; Jordan-Sciutto et al., 2001; Ranganathan and Bowser, 2003; Höglinger et al., 2007; Akay et al., 2011; Zyskind et al., 2015). Thus, determination and maintenance of cell fate by E2F and PPs is a key feature underlying both tissue homeostasis and disease phenotypes.

Mice deficient in only p107 or p130 suffer much less severe phenotypes than pRb knockouts, and are viable and fertile (Cobrinik et al., 1996; Lee et al., 1996). However, a deeper analysis of these models, particularly for p107, revealed key functions for the E2F/PP pathway in not only differentiation, but also direct regulation of stem and progenitor cell maintenance. For example, p107 is required in the developing and adult forebrain to both promote neuronal differentiation and limit neural precursor cell (NPC) expansion (Vanderluit et al., 2004, 2007), and for proper lineage commitment in adipose stem cells (Scimè et al., 2010; De Sousa et al., 2014).

**FIGURE 1:**
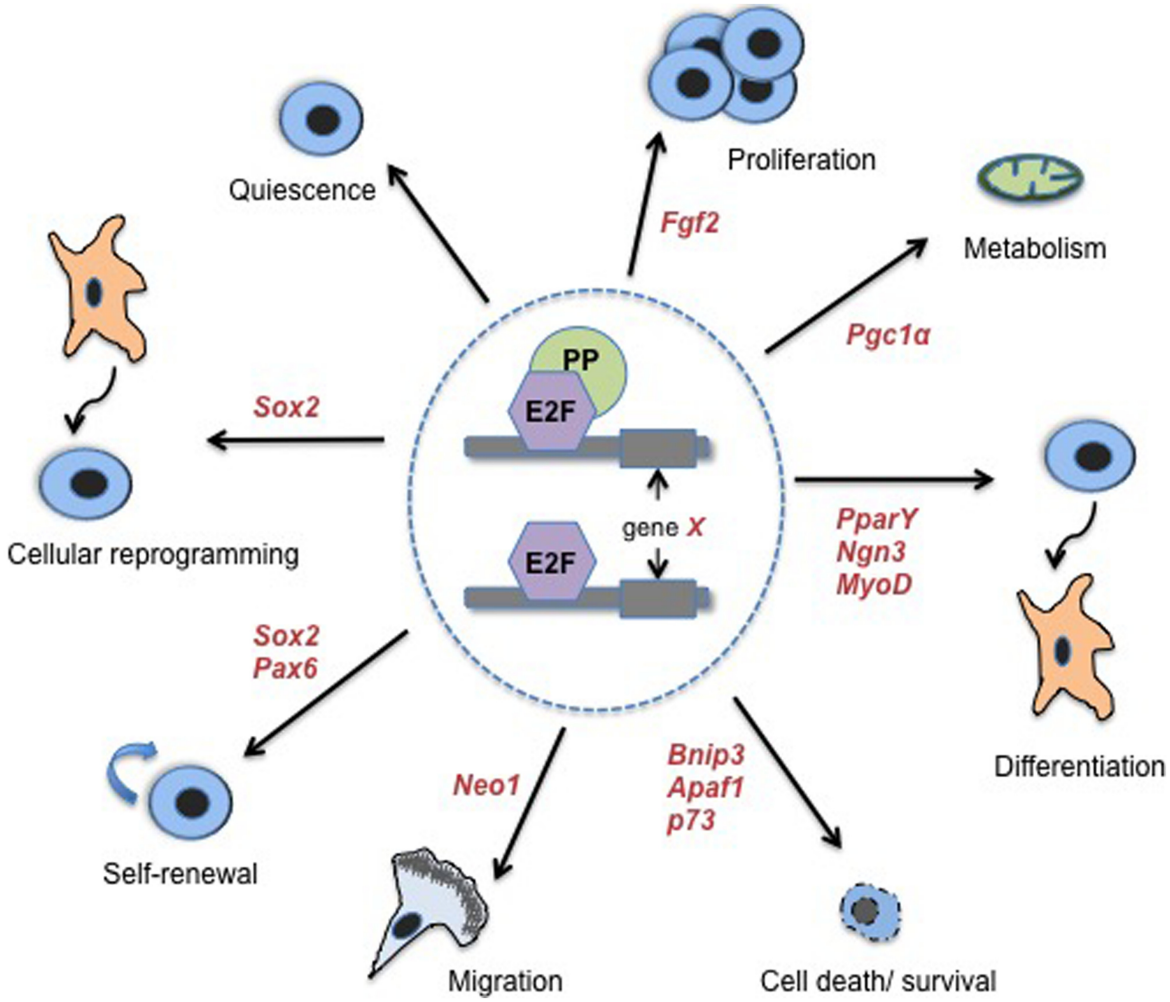
**E2Fs/PPs control diverse classes of cell fate regulatory processes and associated genes**. Transcriptional regulation of non-classical, cell cycle-independent, genes by E2Fs and PPs has been associated with a number of biological processes that impact stem cell fate. These processes include: quiescence, proliferation, metabolism, differentiation and lineage choice, cell death and survival, migration of newly committed cells, stem cell self-renewal, and cellular reprogramming (specifically, reprogramming of fibroblasts to pluripotent SCs). Where examples are known, select cell fate-associated genes that have been confirmed as functional targets of E2Fs/PPs are indicated in red italicized font. References for cell death/survival genes are as follows: (Irwin et al., 2000; Moroni et al., 2001; Hershko and Ginsberg, 2004; Tracy et al., 2007). See the main text or **Table 1** for additional references.

Many PP-mediated phenotypes that impact fundamental stem and progenitor cell fate decisions have been shown to be fully or at least partially E2F-dependent, typically due to a clear transcriptional-based mechanism (Chen et al., 2007, 2009a, 2013; McClellan et al., 2007, 2009; Wenzel et al., 2007; Chong et al., 2009a; Shamma et al., 2009; Hu et al., 2012; Rotgers et al., 2014). Additionally, E2F-deficiency alone, even loss of single E2F family members, disrupts cell fate regulation in a number of cell types (McClellan and Slack, 2007; Ruzhynsky et al., 2007; Asp et al., 2009; Chen et al., 2009a, 2013; Chong et al., 2009b; Julian et al., 2013; Suzuki et al., 2014). Thus, it is clear that transcriptional regulation mediated by E2Fs and PPs is an important functional mechanism underlying stem cell fate determination (**Figure 1**).

## Intersection of Cell Cycle Regulation with Cell Fate Control

Cell cycle dynamics are in fact tightly connected with stem cell fate. Cellular differentiation occurs when a primitive, progenitor cell type acquires more specialized functions, and many cell differentiation events are accompanied by changes in proliferation status. For instance, skeletal muscle precursors irreversibly exit the cell cycle once they terminally differentiate into myocytes (Bischoff and Holtzer, 1969; Nadal-Ginard, 1978; Olson, 1992), and slow-dividing stem cells of the intestinal crypt give rise to transit-amplifying precursors that proliferate quickly before undergoing terminal differentiation into one of the intestinal cell types [reviewed in Potten and Loeﬄer (1990)]. The self-renewal or differentiation potential of pluripotent stem cells is tightly linked to cell cycle phase, where G1 phase cells are poised for differentiation (Sela et al., 2012; Chetty et al., 2013; Singh et al., 2013). Similarly, NPCs lengthen their G1 phase and shorten S phase upon commitment to differentiation (Takahashi et al., 1995; Calegari et al., 2005; Arai et al., 2011), and disrupted cell cycle dynamics severely affect the balance between NPC populations and newly born neurons in the brain (Lange et al., 2009; Lim and Kaldis, 2012). Gain- or loss-of-function studies have revealed key roles for cell cycle proteins in controlling cellular processes and cell fate decisions that influence cortical development, neurogenic output and the number and behavior of neural stem and progenitor cells (these studies will be discussed further below). As cell cycle regulation and cell fate decisions are so closely interconnected, one might therefore argue that the non-canonical activities of E2Fs/PPs that have now been identified in stem and progenitor cells are a secondary consequence of cell cycle control.

One potential mechanism by which E2F- and PP-dependent regulation of cell cycle genes may indirectly influence cell fate decisions is through alteration of cell cycle kinetics. The “cell cycle length hypothesis” postulates that the time spent by tissue progenitors in the G1 phase might increase the ability of these cells to respond to differentiation cues, for example the response to certain morphogens (Lange and Calegari, 2010). In this scenario, PPs would control cell fate in cooperation with E2F factors by silencing their canonical cell cycle target genes, blocking S-phase entry and lengthening the G1 phase. This mechanism has been proposed for adipose and neural precursors (Calegari and Huttner, 2003; De Sousa et al., 2014). However, a number of recent studies have highlighted direct, cell cycle-independent roles for E2Fs in controlling many of these diverse processes. Phenotypic studies in a number of tissues have offered clear evidence that E2Fs and PPs can regulate cell fate decisions that impact stem cell maintenance and differentiation without simultaneously affecting cell cycle dynamics (Ceol and Horvitz, 2001; McClellan et al., 2007; Vanderluit et al., 2007; Chen et al., 2009a; Chong et al., 2009b; Wenzel et al., 2011; Julian et al., 2013; Kareta et al., 2015). A primary, fundamental role for E2Fs outside of cell cycle regulation is further supported by findings in the nematode *Caenorhabditis elegans*, where the E2F, DP and pRb orthologs *elf-1*, *dpl-1* and *lin-35*, are essential for fertility by controlling differentiation of precursor cells during vulval development by antagonizing Ras-MAPK signaling, as opposed to regulating proliferation (Ceol and Horvitz, 2001; Myers and Greenwald, 2005).

A pivotal question remains, however, in determining whether E2Fs and PPs can truly regulate cell fate processes in a direct, cell cycle-independent manner. As the basic functional role of E2Fs is to transcriptionally regulate gene expression, it is important to establish whether they can directly regulate expression of genes that control cell fate processes.

## Unbiased Identification of E2f Target Genes Suggest a Widespread Transcriptional Role in Cell Fate Determination

Despite the extensive and ever increasing evidence of a fundamental biological role for E2F and PPs in stem cell fate control, the underlying cellular mechanisms are only beginning to be clarified. As concerted E2F/PP activity ultimately affects transcriptional regulation of E2F target genes, it is highly likely that the genes that are bound and regulated by E2Fs are pivotal elements of how this pathway controls cell fate decisions. A range of potential scenarios exist, however, whereby E2Fs may have the capacity to regulate genes directly involved in cell fate regulation, or these effects may instead be indirect, caused by secondary effects of cell cycle gene expression and changes in cell cycle dynamics. Understanding these mechanisms at the gene regulatory level is therefore paramount to determining the true nature and extent of E2F/PP function in stem cell biology.

### Early Identification of E2F and PP Target Genes

Among the first genomic studies to take a global look at E2F targets were those employing DNA microarrays to perform gene expression profiling after gain- or loss-of-function of E2F and PP family members. One serious limitation of this approach stems from its inability to distinguish direct and indirect gene regulatory relationships (Ishida et al., 2001; Kalma et al., 2001; Markey et al., 2002; Polager et al., 2002; Stanelle et al., 2002; Vernell et al., 2003; Blais and Dynlacht, 2007). For this reason, experiments of chromatin immunoprecipitation (ChIP) coupled to promoter DNA microarray hybridization were undertaken. The earliest studies used microarrays with long, PCR-generated probes limited to a subset of known genes, typically focused on promoter regions of cell cycle-related genes. These investigations were instrumental in reaffirming previous findings that PPs and E2Fs directly regulate a large cohort of genes associated with proliferative control (Cam and Dynlacht, 2003; Blais and Dynlacht, 2004, 2007; Bracken et al., 2004; Dimova and Dyson, 2005). These canonical target genes include those encoding: key cell cycle regulators (e.g., Cyclin proteins, E2Fs themselves), nucleotide synthesis and DNA replication enzymes (e.g., TK, DHFR, DNA polymerase alpha), DNA repair proteins (e.g., RAD51, the Fanconi anemia proteins), and proteins involved in chromosome organization and segregation (e.g., histones, HMG1, SMC proteins).

This canonical view of E2F-dependent regulation of cell cycle associated genes has come from studies carried out not only in human or rodent cells, but also in flies, nematodes, and plants (Dimova et al., 2003; Stevaux et al., 2005; Kirienko and Fay, 2007; Hirano et al., 2008; Acharya et al., 2012; Korenjak et al., 2012; Kudron et al., 2013). Thus, the role of the E2F/PP signaling node as a key cell cycle regulator, as well as the basic mechanisms of gene regulation by E2Fs and PPs, has deep evolutionary roots.

### E2Fs and PPs as Widespread Regulators of Genes Associated with Stem Cell Fate

Two key advances have contributed to changing the way we now look at the degree of functional diversity of the E2F/PP pathway. First, rapid technological advances in systems biology have increasingly allowed us to perform larger, genome-wide scale screens that are less biased and more likely to provide a comprehensive view of transcription factor targets than the earliest studies of E2F/PP target genes. Second, screens have been performed in a larger diversity of cell types, and in various cell differentiation paradigms, going beyond fibroblasts and cancer cell lines. The data gathered from these studies have revealed that E2Fs and E2F/PP complexes target the promoters of numerous genes with a much broader range of functional associations than was originally perceived, not only the canonical set of cell cycle genes, many of which directly instruct key cell fate decisions. The unbiased identification of E2F/PP target genes, together with the analysis of genetic knock-out animal models, has revealed an incredible diversity of function for the E2f and PP families that cannot be fully appreciated solely with the classical cell cycle regulatory model.

In mammalian cells, large-scale ChIP-chip and ChIP-Seq analyses of E2F target genes that have been performed to date are predominantly focused on identifying E2F1 and E2F4 binding sites, and have been reported in a relatively limited panel of cultured and immortalized cell types (Conboy et al., 2007; Xu et al., 2007; Lee et al., 2011). Unbiased identification of genes bound by E2F3 (including both the E2F3a and E2F3b isoforms) have to our knowledge been reported to date only in C2C12 myoblasts and myotubes (Asp et al., 2009), in mouse embryonic fibroblasts (von Eyss et al., 2012) and in primary murine NPCs (Julian et al., 2015). E2F3a&b, however, exhibit pivotal roles in a number of tissue systems and cellular processes, including gross mammalian embryonic development (Tsai et al., 2008), neurogenesis (Chen et al., 2007; McClellan et al., 2007, 2009; Julian et al., 2013), myogenesis (Asp et al., 2009), Sertoli cell maturation and survival (Rotgers et al., 2014), and maintenance of trophoblast stem cells (Wenzel et al., 2007). Given its broad functional roles, unbiased identification of E2F3 target genes in a greater diversity of cell types will therefore be greatly informative of the conserved and possible tissue-specific mechanisms by which E2F/PPs regulate cell fate decisions.

Despite the relatively limited data currently available, genome-wide DNA binding studies have been instrumental in establishing novel cellular functions for the E2F/PP pathway. Furthermore, they have significantly expanded both the cell cycle-independent roles in which this pathway is implicated, as well as the extent to which it is thought to be integrated transcriptionally in each of these functions. These studies have revealed that E2Fs bind to the regulatory regions of not 100s, as was our previous understanding, but 1000s of genes, in a relatively consistent manner across cell types. Whereas this pathway has been broadly implicated in the regulation of genes involved in not only cell cycle control, but also apoptosis, development, and differentiation for some time (Müller et al., 2001), genome-wide analyses are now demonstrating that E2F factors are in fact poised to control a large network of often 100s of genes involved in each of these biological functions. Additionally, recent studies that have expanded analyses to identify genes bound or regulated at the expression level by E2F/PPs outside of cancerous and immortalized cell lines, specifically in pluripotent, epidermal, muscle and neural stem cells (Asp et al., 2009; Lorz et al., 2010; Yeo et al., 2011; von Eyss et al., 2012; Kareta et al., 2015) have revealed large groups of target genes involved in many specialized functions that influence cell fate. These functions broadly include the regulation of cellular metabolism, quiescence, stem cell self-renewal, and tissue-specific differentiation programs. These findings, and the large number of potential target genes uncovered for each process, suggest a widespread transcriptional role for E2Fs and PPs in stem cell fate regulation.

## Functional Evidence that E2Fs and PPs Control Transcription of Cell Fate-Associated Genes

Functional assays and analyses of genetic mouse models have provided important biological confirmation that E2Fs and PPs can indeed affect cell fate outcomes in stem and progenitor cells by transcriptionally regulating genes that directly control cell fate processes. E2F and PP family members have been implicated as important biological regulators of a number of processes that impact stem and progenitor cell fate, including: death and survival, quiescence, self-renewal, proliferation, differentiation, and migration (**Figure 1**). While classical cell cycle control is implicated in some processes, such as quiescence (Sage et al., 2003; Lorz et al., 2010; Andrusiak et al., 2013), many genes that have direct, seemingly cell cycle-independent roles in cell fate regulation have been validated as true functional E2F/PP targets (**Figure 1** and **Table 1**). Although the number of such validated genes is currently limited, considering the large number of potential target genes uncovered by genome-wide analyses, the findings that have been made provide important proof of concept that this cell fate regulatory mechanism is important across multiple cell lineages.

**Table 1:** Listed here are the stem and progenitor cell populations for which E2Fs and PPs have demonstrated cell fate regulatory roles.

Stem cell population	E2F and PP factors implicated	Cell fate process affected	Target gene(s)	Reference (for target genes)
Pluripotent SC	pRb, p107, p130, E2f2, E2f4	Self-renewal, Reprogramming to pluripotency, Survival	Sox2**	Yeo et al. (2011), Li et al. (2012), Kareta et al. (2015)
Neural and retinal precursors	pRb, p107, E2f1, E2f2, E2f3, E2f4	Self-renewal, Proliferation, Differentiation Migration, Survival	*Sox2*, *Pax6*, Notch and Shh pathways, Fgf2, Dlx1, Dlx2, Neo1, Nrp1**	Vanderluit et al. (2004, 2007), Jiang et al. (2007), McClellan et al. (2007, 2009), Ruzhynsky et al. (2007), Andrusiak et al. (2011), Ghanem et al. (2012), Julian et al. (2013, 2015)
Myoblast	pRb, p107, E2f1, E2f3, E2f4	Proliferation, Differentiation, Survival	MyoD**	Asp et al. (2009)
Hematopoietic SC	pRb, p107, p130, E2f8	Quiescence, Expansion, Differentiation		
Adipogenic progenitor	p107, E2f1, E2f4	Proliferation, Differentiation	*PPARγ*	Fajas et al. (2002b)
Osteoblasts	pRb, E2f1	Differentiation	*Alpl*, *Bglap*	Flowers et al. (2013)
Liver oval SC	pRb, E2f1	Quiescence, Expansion	Notch pathway	Viatour et al. (2011)
Pancreatic/endocrine SCs	E2f1	Proliferation, Differentiation	*Ngn3*	Kim and Rane (2011)
Trophoblast SC	pRb, E2f3, E2f7, E2f8	Proliferation, Differentiation, Survival		
Spermatogonial SC	pRb	Self-renewal		

Also indicated are the specific E2F and PP factors that have been implicated functionally, as well as the cell fate regulatory process known to be controlled by E2F/PPs, in each stem/progenitor cell type. Where examples are known and have been functionally validated, we give key examples of direct cell fate regulatory genes that are transcriptional targets of E2Fs/PPs. The references listed are specific to the target genes given. **Indicates that genome-wide analyses (represented among the references) have uncovered additional putative target genes in that cell type. The cell fate process ‘expansion’ encompasses the potential for both proliferation and/or self-renewal.

### Regulation of Genes that Promote or Inhibit Differentiation

A number of genes that directly control progenitor cell commitment to differentiation or lineage choice have been validated as biologically relevant E2F/PP target genes. For instance, E2f1 stimulates expression of the Peroxisome proliferator-activated receptor PPARγ in adipogenic progenitors to promote their expansion, while E2f4 conversely represses PPARγ expression to limit expansion and promote adipocyte differentiation (Fajas et al., 2002b). Functional analysis of myogenesis, along with direct identification of the genes both bound and regulated at the mRNA level by E2fs in proliferating and differentiating myoblasts revealed that E2f3b is required to repress expression of key myogenic factors, such as MyoD, during differentiation (Asp et al., 2009). Furthermore, E2f1 stimulates pancreatic differentiation by activating expression of the Ngn3 promoter in embryonic endocrine precursors (Kim and Rane, 2011). In the CNS, regulation of the neurogenesis and migration related genes *Dlx1/Dlx2* and *Neo1* (Neogenin) are linked to pRb and E2f-mediated control of interneuron specification and neuronal migration during development (Andrusiak et al., 2011; Ghanem et al., 2012). E2F/PP-mediated transcriptional regulation of factors that potentiate differentiation has also been linked to tumorigenesis, where E2f1-mediated transcription of PPARγ and Fatty acid synthase (Fasn) drives proliferation and survival of medulloblastoma tumors (Bhatia et al., 2012; Bhasin et al., 2013), and activation of multiple Notch pathway genes by E2fs serves to limit tumor expansion in hepatocellular carcinoma (Viatour et al., 2011).

### Regulation of Stem Cell Maintenance Genes by E2Fs and PPs

Recent studies have substantiated a direct transcriptional role for E2Fs and PPs in not only the regulation of differentiated cell fates, but also in the control of stem cell self-renewal and proliferation. Studies of E2F/PP biological function and associated target genes in the CNS in particular have significantly increased our understanding of how E2F/PP activity can impact stem cell function. In the adult CNS, loss of E2f1 leads to a reduction of neural stem and progenitor cell divisions in the proliferative zones, resulting in reduced hippocampal neurogenesis (Cooper-Kuhn et al., 2002). In the retina, pRb and E2f3a together control differentiation, specifically of starburst amacrine cells (Chen et al., 2007). In the developing telencephalon, however, p107 and E2f3a/b regulate the balance between NPC maintenance, self-renewal and differentiation, and these activities are strongly associated with transcriptional regulation of the core stem cell self-renewal/maintenance genes *Sox2* and the Notch/Hes pathway (Vanderluit et al., 2004, 2007; Julian et al., 2013). E2f4 also promotes neural stem cell self-renewal, and this has been linked to regulation of the Sonic Hedgehog (Shh) pathway (Ruzhynsky et al., 2007), another core regulator of neural stem cell maintenance. Additionally, p107 and E2f3 control NPC proliferation in the developing brain through regulation of the fibroblast growth factor *Fgf2* (McClellan et al., 2009).

These studies reveal a highly dynamic role for PPs and E2F factors in CNS development and homeostasis. Furthermore, they heavily implicate transcriptional regulation of non-canonical, cell fate-associated genes as a driving mechanism behind E2F/PP-dependent function in stem and progenitor populations. In line with the situation in the CNS, E2F/PP activity has also recently been implicated in controlling the self-renewal potential of pluripotent ESCs in mammals. Transcriptomic and transcription factor motif analyses in human ESCs suggested a fundamental role for E2F factors in the self-renewal of pluripotent stem cells (Yeo et al., 2011), which has been confirmed functionally (Conklin et al., 2012; Suzuki et al., 2014). This study suggested that E2Fs were highly integrated in the self-renewal network, and our recent bioinformatics analysis of E2f3 and E2f4 direct binding sites in murine NPCs confirmed that E2fs do indeed bind to an extensive network of genes fundamental for self-renewal and stem cell function (Julian et al., 2015).

This identification of E2Fs as important regulators of stem cell self-renewal and associated core regulatory genes has important implications not only for tissue homeostasis and development, but also the tumorigenic or tumor suppressive role of E2F and PP factors. An interesting possibility is that cancer may arise due to a loss of the ability to control expression of stem cell self-renewal genes, in addition to *bona fide* cell cycle genes. Supporting this assertion, two recent studies demonstrated a pivotal role for E2F/PP complexes in inhibiting cellular reprogramming to pluripotency. Specifically, two forms of transcriptional repressive complexes, pRb/E2f as well as p130/E2f4 in complex with the Cyclin-dependent kinase inhibitor p27, were shown to function as inhibitory blocks to reprogramming (Li et al., 2012; Kareta et al., 2015). Intriguingly, the underlying mechanisms were independent of cell cycle control, but due to transcriptional repression of Sox2 expression during the reprogramming process. Furthermore, the ability of pRb/E2f to repress Sox2 expression appeared to be a critical tumor-suppressive mechanism (Kareta et al., 2015). These studies, together with the findings of E2f-dependent *Sox2* regulation in NPCs (Julian et al., 2013), establish the E2F/PP regulatory node as an essential regulator of one of the most fundamental stem cell identity genes, importantly in two primary cell types that rely heavily on Sox2 for maintenance of their stem cell pool.

### Regulation of Genes that Control Cell Death and Survival

A role for E2Fs and PPs in mediating cell death and/or survival has been functionally described in many lineages. Although this biological role has been known for some time, the mechanisms affecting cell death in stem and progenitor cells due to deregulation of E2Fs or PPs is not fully clarified. Nevertheless, p53-dependent mechanisms have been highly implicated, and while recent evidence suggested a non-transcriptional role for pRb in apoptotic induction (Hilgendorf et al., 2013), a number of genes that are involved in both the mitochondrial apoptotic signaling cascade as well as autophagy regulation have been demonstrated as downstream or direct targets of E2Fs and/or PPs (Hiebert et al., 1995; Sherr, 1998; Irwin et al., 2000; Moroni et al., 2001; Nahle et al., 2002; Vorburger et al., 2002; Hershko and Ginsberg, 2004; Hershko et al., 2005; Tracy et al., 2007; Ianari et al., 2009; Conklin et al., 2012; Bertin-Ciftci et al., 2013; Sung et al., 2013; Benson et al., 2014). Furthermore, a specific biochemical interaction between pRB and E2F1 is required for regulation of both E2F1-induced apoptosis and expression of E2F-dependent apoptotic genes (Dick and Dyson, 2003; Julian et al., 2008; Carnevale et al., 2012), strongly suggesting that transcriptional regulation by E2F/PP is a primary mechanism by which this pathway controls cell death.

### Transcriptional Regulation of Metabolism by E2Fs/PPs

In addition to cell cycle dynamics and execution of stem cell-specific gene regulatory networks, a plethora of recent work has revealed an essential role for metabolic adaptations in driving the stem cell state. Specifically, it has become clear that stem cells inhibit oxidative metabolism and depend on metabolic pathways that rely heavily on glycolysis for energy production (Folmes et al., 2013; Ochocki and Simon, 2013). Given this knowledge, recent findings demonstrating a requirement for E2F and PP factors to both inhibit oxidative phosphorylation/promote glycolytic pathways in muscle and adipose tissue and to repress expression of genes associated with oxidative metabolism, such as PGC1α, are particularly intriguing (Scimè et al., 2010; Blanchet et al., 2011). While the functional relevance of E2F/PP-dependent regulation of core metabolism genes has not been investigated in stem cell populations, genome-wide studies have identified numerous metabolism-related genes as putative E2F targets (Asp et al., 2009; Yeo et al., 2011; Julian et al., 2015). Importantly, these discoveries in addition to others discussed here, place E2Fs and PPs as pivotal transcriptional regulators of multiple essential biological processes and regulatory programs that control stem cell fate decisions (**Figure 1**).

## Mechanisms of Cell Fate Gene Regulation by E2F/PPs

**FIGURE 2:**
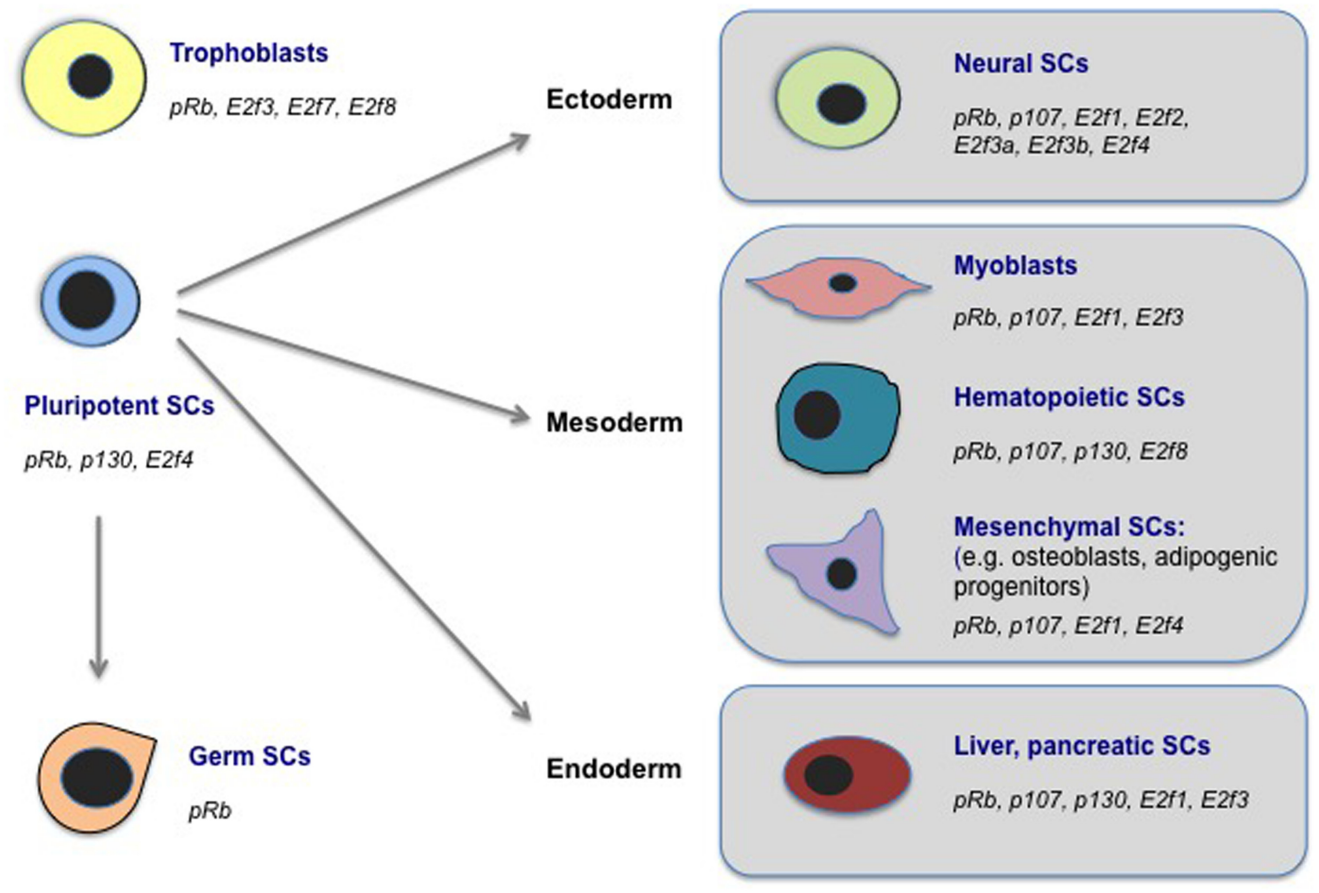
**E2Fs and PPs regulate cell fate in diverse stem cell populations**. Here, we highlight examples of mammalian stem cell (SC) populations in which biological roles for E2Fs and/or PPs in cell fate regulation, outside of classical cell cycle control, have been documented. These cell types include SC populations representing all three embryonic germ layers (ectoderm, mesoderm, endoderm), as well as pluripotent SCs (in ESCs as well as during cellular reprogramming to iPSCs), germ (spermatogonial) SCs, and extra-embryonic trophoblasts. We indicate the E2F and PP factors that have been implicated in cell fate regulation in each SC type. See the main text or **Table 1** for references.

As discussed above, the cell fate-associated processes with which E2Fs and PPs have been functionally implicated are diverse. In mammals, the PP and E2F families are now known to impact cell fate determination in many lineages. This includes neuronal, mesenchymal, hematopoietic, muscle, intestinal, mammary gland, liver, trophoblast, spermatogonial, and pluripotent stem and progenitor cells [for a thorough review on many of these lineages, see (Daria et al., 2008; Viatour et al., 2008; Ouseph et al., 2012; Sage, 2012; Hu et al., 2013; Yang et al., 2013; Suzuki et al., 2014; Rotgers et al., 2014; Kareta et al., 2015]. Thus the current evidence suggests that these proteins play instructive roles in stem and progenitor cell types that encompass all embryonic germ layers, as well as extra-embryonic, germ cell, and pluripotent lineages (**Figure 2** and **Table 1**). While our understanding of the full extent of stem and progenitor lineages that are affected by E2F/PP fate control is incomplete, these findings suggest that E2F/PP-dependent mechanisms are pervasive and perhaps fundamental for stem cell fate control. Additionally, they suggest that E2F and PP factors may have the capacity to regulate unique classes of cell fate regulatory genes in different tissue types. While the extent of tissue-specific gene regulation by E2F/PPs is poorly understood, emerging data suggests that it is extensive and likely to involve multiple mechanisms to influence target gene selection.

### Regulation of Tissue-Specific Genetic Networks

A long-standing theory proposed to explain the ability of cell cycle regulators to potentiate tissue-specific differentiation programs, on a biological level, is that PPs interact with transcriptional co-factors other than E2Fs that are unique to specific tissues and that regulate tissue-specific target genes. Indeed, pRb has been shown to complex with transcription factors other than E2Fs in a manner that affects progenitor cell differentiation, one prominent example being its interaction with Runx2 in the osteoblast lineage to regulate the expression of osteoblast-specific genes (Thomas et al., 2001). An interaction between pRb and MyoD in muscle cells has also been reported (Gu et al., 1993), but it is likely that the interplay between the PP and the master regulator of myogenesis occurs indirectly, through competition for binding to the transcriptional co-repressor HDAC1 (Mal et al., 2001; Puri et al., 2001). pRb has also been shown to repress adipocyte differentiation by interacting with PPARγ and recruiting HDAC1 to its target promoters (Fajas et al., 2002a), and to stimulate adipogenesis by interacting with CEBP transcription factors (Chen et al., 1996). Additionally, stabilization of the homeobox protein Pdx1 through a direct interaction with pRb is necessary for embryonic pancreas development and adult β-cell function (Kim et al., 2011). It is likely that more tissue-specific interactions of this kind will be discovered as this line of investigation progresses.

**FIGURE 3:**
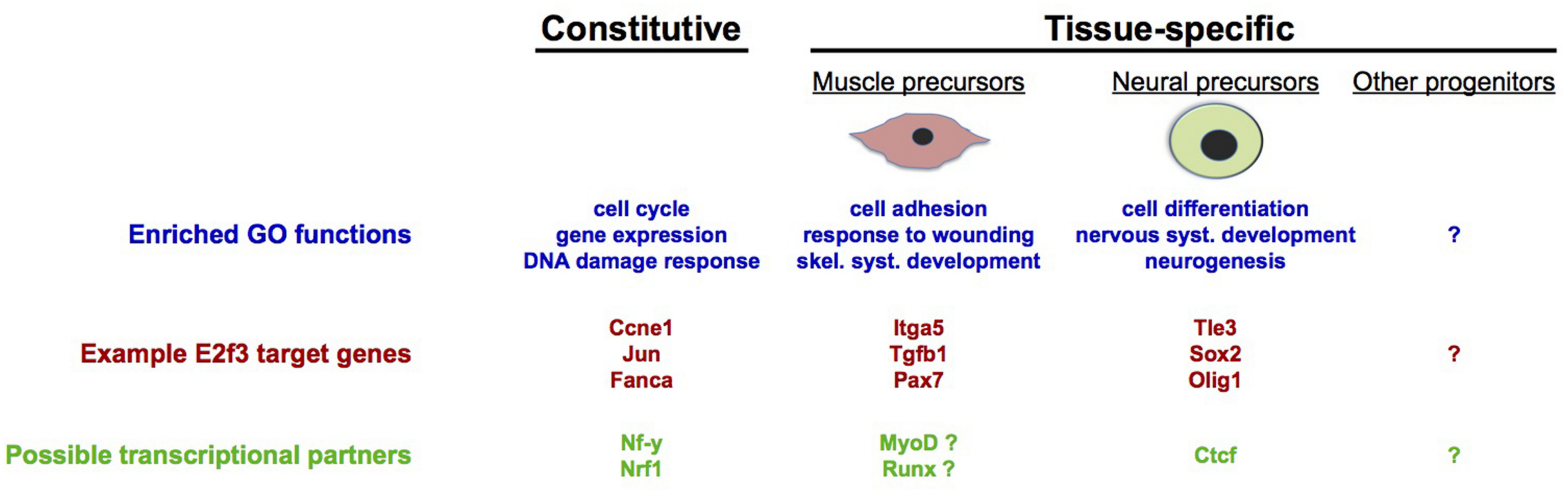
**Tissue-specific gene regulation by E2F3**. Genome-wide analyses in precursors of skeletal muscle and neurons have revealed the existence of tissue-specific target genes, as well as genes that are likely to be constitutively regulated in most tissues. Constitutive and tissue-specific E2f3 targets are enriched in different functional categories (gene ontologies), with cell cycle-related functions being most represented by constitutive targets of E2f3. Additionally, gene promoter sequence analyses suggest that E2f3 may cooperate with different transcriptional regulators, depending on the cell type: with Nrf1 (Cam et al., 2004), Sp1 (Blais et al., 2002), and NF-y (Caretti et al., 2003; Elkon et al., 2003; Zhu et al., 2004) for constitutive cell cycle target genes, with Ctcf in neural precursors (Julian et al., 2015) and with MyoD and Runx in myoblasts [unpublished analyses performed using Whole Genome rVista (Dubchak et al., 2013)].

Independently from these possibilities, however, the evidence that is now emerging from a deeper analysis of E2F target genes in individual cell types suggests that a prominent mechanism by which cell cycle regulators control tissue-specific cell fate decisions is through E2F-dependent regulation of networks of cell fate regulatory genes that are specific to that lineage. Due to the accumulating evidence that E2Fs and PPs can control cell fate processes without affecting cell cycle dynamics, and the expanding number of direct E2F target genes that control tissue-specific stem cell fate decisions (Fajas et al., 2002b; Ruzhynsky et al., 2007; Asp et al., 2009; McClellan et al., 2009; Andrusiak et al., 2011; Ghanem et al., 2012; Li et al., 2012; Julian et al., 2013; Kareta et al., 2015), we anticipate that further investigations into the genome-wide binding sites of E2Fs in different cell types will solidify the hypothesis that E2Fs target large networks of tissue-specific target genes. Speaking to this, a recent study identified extensive tissue-specificity in the binding sites of E2F and PP orthologs in germline and somatic cell populations in *C. elegans* (Kudron et al., 2013). In mammalian cells, comparative analysis of E2f3-bound gene promoters in murine NPCs and myoblasts showed that while cell cycle-related target genes are common to both cell types, there is a large degree of tissue-specificity among E2f3 target sites, specifically at those genes involved in differentiation and development-related processes (Julian et al., 2015; **Figure 3**). To understand how widespread this phenomenon is, it is imperative that systematic analysis of genome-wide E2F binding sites, and corresponding gene expression analyses, be performed in a much more expansive group of mammalian tissues and primary cell types.

### Diverse Transcriptional Roles of E2Fs and E2F/PP Complexes

Multiple lines of evidence suggest that E2F/PP-mediated regulation of cell fate-associated genes does not closely follow the canonical view that E2Fs1-3a are predominantly transcriptional activators, and the remaining E2Fs are predominantly repressors that function in cooperation with a PP. First, the fact that genomic binding studies have identified both ‘activator’ and ‘repressor’ E2Fs at seemingly active promoters, in multiple stem and progenitor cell types, does not support this canonical view (Asp et al., 2009; Yeo et al., 2011). A number of observations from single gene-focused analyses have further revealed that E2F transcriptional function is more complex than the canonical model suggests. Unexpectedly, the E2f3-mediated regulation of Sox2 in NPCs was paradoxically found to be dependent on a transcriptional activation role for E2f3b and a repressive role for the classical ‘activator’ E2f3a (Julian et al., 2013). Additionally, this repressive role for E2f3a appears to function in concert with p107, an atypical binding partner for E2f3a as it is was thought to only form PP-containing complexes with pRb. Interestingly, E2f3a has also been shown to mediate repression in starburst amacrine cells in the retina, this time through collaboration with pRb (Chen et al., 2007). Additionally, a role for pRb/E2f1 complexes in gene activation has been demonstrated at select genes involved in osteogenic, adipogenic, and myogenic differentiation (Flowers et al., 2013), while alternatively, pRb-independent gene repression by E2f3b, across a large panel of genes, has also been observed (Asp et al., 2009).

Intriguingly, the simultaneous identification of biologically functional E2f3b activator and E2f3a/p107 repressor complexes at the same target gene, Sox2, in proliferating NPCs, suggests that a homeostatic level of E2F and PP family members is required to ensure proper regulation of at least some target genes. By this mechanism, proper biological function would be dependent on finely tuned transcriptional regulation by E2F and PPs, as opposed to strict ‘on/ off’ activation or repression by specific transcriptional complexes. The requirement for opposing regulation of Sox2 by E2fs/PPs to regulate the balance between NPC maintenance and differentiation (Julian et al., 2013), as well as findings that both pRB over-expression and deficiency in human ESCs induces cell cycle arrest and death (Conklin et al., 2012), and that both loss or gain of E2f expression drives survival defects in retinal progenitors (Chen et al., 2009a; Chong et al., 2009b), are testaments to this possibility.

Together, these findings paint a more malleable picture of transcriptional regulation by E2Fs and PPs than what the canonical model suggests, where the potential combinations of E2F and PP factors at DNA sites and their resulting transcriptional effects are in fact diverse and not clearly predictable. The complexes that are formed and their transcriptional effects at cell fate regulatory target genes are likely to be influenced by a number of factors, including cell type- and state-specific expression profiles of E2F and PP family members and additional co-factors, as well as the chromatin environment that surrounds particular E2F-bound sites. Thus, it is likely that transcriptional regulation by E2Fs and E2F/PP complexes can vary significantly at different genomic sites and in different cellular states.

### Transcriptional Co-Factors and E2F Target Gene Selection

Given the association of E2F and PP factors with cell fate regulation in what is now known to be a considerably broad range of cell types, especially with the potential tissue-specificity of this phenomenon, understanding how these proteins are able to physically discern between their canonical cell cycle regulatory genes and their non-canonical targets becomes an important question. The fact that many E2F/PP-dependent cell fate regulatory functions can be functionally separated from cell cycle control suggests that these proteins are recruited to ‘cell cycle’ and ‘cell fate’ genes with the help of different transcriptional partners.

While a prospective analysis of potential E2F co-factors that may specifically regulate cell fate genes has not been reported, two co-factors for E2F3, to date the most highly implicated E2F family member in cell fate control, have been identified. Specifically, the E-box transcription factor TFE3 has been shown to interact uniquely with E2F3 through its marked box domain and to regulate proliferation and the expression of select genes in cooperation with E2F3 (Giangrande et al., 2003; Nijman et al., 2006). Additionally, a recent study demonstrated that the SNF2-like helicase protein HELLS interacts with E2F3 in the context of tumorigenesis to induce cell cycle entry and proliferation, and that the two appear to synergistically activate select target genes (von Eyss et al., 2012). These studies implicated TFE3 and HELLS as E2F3 co-factors largely in the context of proliferative control. It is possible that these interactions have the same functional consequence in all cell types; however, it may also be the case that TFE3 and HELLS are important factors in the recruitment and/or activity of E2F3 to cell fate regulatory genes in stem cell populations. As the functional implications and conservation of these interactions have not been extensively characterized, this possibility warrants further investigation.

In addition to potential co-factors that may recruit E2Fs to target sites, recruitment of E2Fs to target genes can also be regulated by mechanisms that compete for their ability to bind DNA. For instance, the Cyclin-dependent kinase CDK5 is a potent cell cycle suppressor in post-mitotic neurons (Zhang et al., 2008), and the underlying mechanism is due to the ability of a CDK5-p35 complex to directly bind E2F1, consequently disrupting the ability of E2F1 to interact with DP1 on DNA at various cell cycle-related genes (Zhang et al., 2010). As enzymatically active CDK5 is restricted to post-mitotic neurons, studies have largely focused on determining its function in this cell type. However, the mechanism described here is not dependent on enzymatic activity, and since CDK5 is broadly expressed (Tsai et al., 1993) this unique E2F regulatory mechanism may be important in other cell types. It is unclear at the moment if such a mechanism may similarly contribute to the regulation of cell fate regulatory genes by E2Fs in neural cells or other lineages, but it is a promising possibility that this or a similar mechanism contributes to E2F target gene specificity.

Another intriguing possibility is that interaction between E2Fs and PPs with enhancer regions may underlie the ability of these proteins to bind to potentially unique sets of cell fate associated genes in different cell types, as enhancers are key mediators of cell type-specific gene regulation. The recent finding that a significant proportion of E2F4 binding sites are directly associated with enhancers lends credence to this idea (Lee et al., 2011). Furthermore, gene promoter sequence analysis of E2f3-bound promoter sites has identified a few select factors that may discern common and tissue-specific E2f target sites in NPCs and myoblasts (Julian et al., 2015; unpublished data; **Figure 2**). Intriguingly, further bioinformatic analyses revealed CTCF as a potential novel co-factor for E2f3 at cell fate genes specifically in NPCs. CTCF is a well-known insulator protein associated with enhancer regions and, as recently demonstrated, with a sub-population of promoter sites (Shen et al., 2012; Phillips-Cremins and Corces, 2013). Given the particular importance of CTCF in neuronal development (Hirayama et al., 2012), enhancer–promoter connections mediated between CTCF and E2F represents a particularly promising mechanism for NPC-specific cell fate gene regulation by E2F/PPs.

As the identity of protein complexes found at enhancer regions and their interactions with promoters is a major mechanism dictating cell type-specific gene expression, this is an exciting finding that suggests E2Fs may influence tissue-specific cell fate control by coordinating enhancer–promoter interactions at key cell fate associated genes. Application of ChIP-Seq to identify truly unbiased genome-wide binding sites of additional E2F factors in a greater diversity of cell types will importantly reveal how widespread this phenomenon is among the E2F family. Coupling this approach with genomic structural analyses, such as Hi-C technology, which allows for identification of chromatin loop domains and associated chromatin marks and binding proteins (Rao et al., 2014), will provide an important perspective on the potential functional implications of these interactions.

## Conclusion and Perspectives

The relatively recent technical progress in systems biology approaches to understanding gene regulation on a genome-wide level has revealed an extensive diversity of function for the classical cell cycle regulatory E2F/PP pathway. We now know that transcriptional regulation of extensive sets of cell fate regulatory genes by E2Fs and PPs is an important regulatory mechanism underlying key cell fate decisions in a number of cell types. Emerging evidence also suggests that the E2F/PP signaling node is able to mediate cell type-specific gene expression programs. While advances over the past few years have greatly expanded our view of the functional importance of transcriptional regulation by E2Fs and PPs, the mechanistic understanding of their role in stem cell fate regulation is in its infancy. We need a better understanding of which stem cell populations rely on E2F/PP activity when making key cell fate decisions, as well as which epigenetic co-factors contribute to gene class and cell type-specific gene expression.

Moving forward it will be important to continue to exploit advances in systems biology approaches that allow for truly genome-wide analyses of transcription factor binding sites in order to understand the full extent of E2F/PP function in stem cell fate control. Correlation of putatively identified target genes with gene expression signatures, co-factor binding, and both two- and three-dimensional chromatin structure will shed important mechanistic insight on the epigenetic role of E2Fs/PPs in cell fate decision making.

An important question for future investigations, which is currently largely unaddressed, is how E2Fs and PPs may regulate cell fate genes in post-mitotic cells. There is extensive evidence that PPs and E2Fs can repress cell cycle entry in post-mitotic cells and that they can participate in the formation of multi-protein repressive complexes in these cell types to repress classical E2F cell cycle target genes [reviewed in Blais and Dynlacht (2007), Dick and Rubin (2013), Herrup (2013)]. It is therefore likely that E2Fs and PPs are important regulators of cell fate-associated genes in post-mitotic cells, in both normal and disease settings, perhaps to repress the stem cell state, or to maintain differentiation and survival. Gaining a clearer understanding of the mechanisms underlying epigenetic cell fate regulation by E2Fs/PPs by addressing these key questions will have important implications in the contexts of tumorigenesis and disease, development, tissue homeostasis, and regeneration.

## Conflict of Interest Statement

The authors declare that the research was conducted in the absence of any commercial or financial relationships that could be construed as a potential conflict of interest.
